# Screening a small molecule library to identify inhibitors of NF-κB inducing kinase and pro-labor genes in human placenta

**DOI:** 10.1038/s41598-018-20147-0

**Published:** 2018-01-26

**Authors:** Bingbing Wang, Nataliya Parobchak, Adriana Martin, Max Rosen, Lumeng Jenny Yu, Mary Nguyen, Kseniya Gololobova, Todd Rosen

**Affiliations:** 1 Department of Obstetrics, Gynecology, and Reproductive Sciences, Division of Maternal-Fetal Medicine, Rutgers Robert Wood Johnson Medical School, New Brunswick, New Jersey USA; 2 Ernest Mario School of Pharmacy, Rutgers University, Piscataway, New Jersey USA

## Abstract

The non-canonical NF-κB signaling (RelB/p52) pathway drives pro-labor genes in the human placenta, including corticotropin-releasing hormone (CRH) and cyclooxygenase-2 (COX-2), making this a potential therapeutic target to delay onset of labor. Here we sought to identify small molecule compounds from a pre-existing chemical library of orally active drugs that can inhibit this NF-κB signaling, and in turn, human placental CRH and COX-2 production. We used a cell-based assay coupled with a dual-luciferase reporter system to perform an *in vitro* screening of a small molecule library of 1,120 compounds for inhibition of the non-canonical NF-κB pathway. Cell toxicity studies and drug efflux transport MRP1 assays were used to further characterize the lead compounds. We have found that 14 drugs have selective inhibitory activity against lymphotoxin beta complex-induced activation of RelB/p52 in HEK293T cells, several of which also inhibited expression of CRH and COX-2 in human term trophoblast. We identified sulfapyridine and propranolol with activity against CRH and COX-2 that deserve further study. These drugs could serve as the basis for development of orally active drugs to affect length of gestation, first in an animal model, and then in clinical trials to prevent preterm birth during human pregnancy.

## Introduction

Preterm birth is arguably the greatest risk facing pregnant women and their newborns, and one of the greatest health problems facing the populations in the United States and countries around the world. It is the leading cause of neonatal morbidity, with two-thirds (67.0%) of all infant deaths in the United States occurring after a preterm delivery. In 2013, the mortality rate for very preterm infants was 88 times greater than for those born at term; even at 32–33 weeks of gestation, the risk for death was nearly 9 times greater than those born full term^1^. The annual societal economic burden associated with preterm birth in the United States was at least $26.2 billion in 2005, or $51,600 per infant born preterm^2^. Despite the tremendous health and economic costs associated with preterm birth, therapies to prevent early deliveries remain largely ineffective and new approaches to this problem are necessary.

The placental endocrine system plays a central role in onset of human parturition^3^. Corticotropin-releasing hormone (CRH), produced by syncytiotrophoblast, may be part of a clock that determines length of human gestation^4–7^. Prostaglandins (PGs), produced by COX-2 in the placenta and fetal membranes, contribute to initiation of both term and preterm labor^8,9^. Recently, we have shown that the non-canonical NF-κB signaling pathway, functioning in human placenta under influence of glucocorticoids and progestins, regulates CRH and COX-2^10–13^.

The non-canonical by NF-κB-inducing kinase (NIK). NIK phosphorylates the inhibitory-κB kinase-α (IKKα) complex that, in turn, phosphorylates the IKB (inhibitory κB) domain of NF-κB2 (p100). p100 is then degraded by the proteasome after its phosphorylation and ubiquitination to release p52^14^. The liberated p52 forms a heterodimer with RelB and subsequently translocates into the nucleus to regulate target genes. Activation of this pathway may be triggered by a subset of TNF receptor family members, and in select tissues like activated B lymphocytes^15^ and neurons^16^, it may be constitutively active. Interestingly, we have found in a previous study, that like plasma cells and select neurons, the non-canonical NF-κB pathway is also constitutively activated in term cytotrophoblast under the persistent influence of glucocorticoid^10^.

We hypothesized that targeting the non-canonical NF-κB pathway and by inhibiting kinases that regulate its activity, would modulate the activity of hormones posited to play a role in human parturition. In an effort to identify specific inhibitors of non-canonical NF-κB activity, and in turn CRH and COX-2, *in vitro* studies were performed on 1,120 drugs in the Prestwick chemical library, a collection of small molecular inhibitors composed of FDA-approved drugs and natural products^17^. We identified 14 small molecule compounds that were specific inhibitors of non-canonical NF-κB activity. 4 of these 14 agents were neither actively transported out of the placenta nor toxic to cultured cytotrophoblast and are candidates for further study *in vivo* as potential therapies to modulate the placental clock, and in turn, potentially prevent some cases of preterm birth. Two agents may be worthy of studying *in vivo* in a non-human primate model.

## Results

### *In vitro* screen for inhibitors of the non-canonical NF-κB pathway

**Figure 1:**
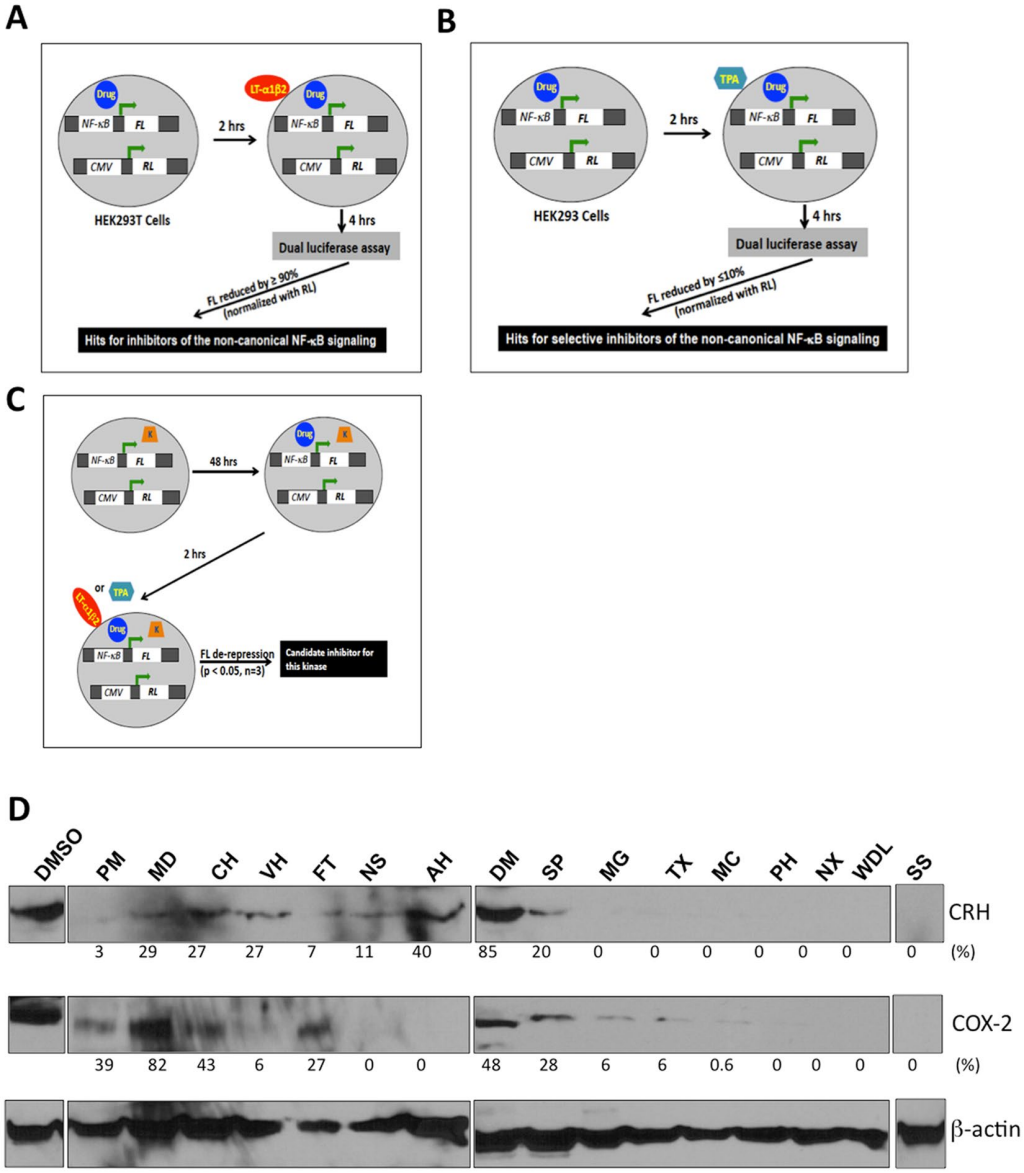
Schematic presentation of strategy of drug screening. (**A**) Initial screening. HEK293T cells were transiently transfected with a mixture of firefly luciferase (FL) reporter and renilla luciferase (RL) reporter vectors. 48 hours later, the transfectants were exposed to individual compound (drug) for 2 hours, and in turn, lymph toxin- a1b2 (LT-α1β2) for 4 hours prior to dual-luciferase assay. Each chemical compound was run in triplicates and the averages of FL and RL in triplicates were obtained, respectively. With normalization to RL, the individual compound with ≥90% of FL inhibitory effect compared with the vehicle control was considered as a hit for inhibition of the non-canonical NF-κB signaling activity. (**B**) Secondary screening. HEK293T cells were transiently transfected with a mixture of vectors as described in (**A**). 48 hours later, the transfectants were exposed to individual hits from the initial screening, and in turn, TPA for 4 hours prior to dual-luciferase assay. Each chemical compound was run in triplicates and the averages of FL and RL in triplicates were obtained, respectively. With normalization to RL, the individual compound with ≤10% of FL inhibitory effect compared with the vehicle control was considered as the specific inhibitor for the non-canonical NF-κB signaling activity. (**C**) Screening of kinase inhibitors. HEK293T cells were transiently transfected with a mammalian expression vector for ectopic expression of an individual kinase (K), which was followed by a mixture of vectors as described in (**A**). Then the transfectants were treated with LT-α1β2 (for NIK and IKKα) or TPA (for IKKβ). Each experiment was run in triplicates. With normalization to RL activity, the individual compound with de-repressed FL activity compared with the empty vector control (p < 0.05) was considered as a candidate for inhibition of this kinase. (**D**) Representative gel pattern and analysis were obtained from 3 independent term placentas by Western blotting with abbreviations described in Table 1. The cropped gels from different Western blot analysis were divided by white space with full-length blots shown in Supplementary Fig. 1. Western blot results were quantified with use of ImageJ. The number below each lane representing relative protein levels of CRH or COX-2 treated with the individual drug relative to DMSO with normalization to β-actin was derived from the following formula: (peak area of CRH or COX-2 of each drug)/(peak area of β-actin of the same lane) of/(peak area of CRH or COX-2 of DMSO)/(peak area of β-actin of the same lane) × 100%. The relative abundance of CRH or COX-2 in the lane of DMSO was considered 100%.

Initial screening of inhibitors of the non-canonical NF-κB activity from the Prestwick Chemical Library was performed in HEK293T cells by using a dual-luciferase reporter system (Fig. 1A). A mixture of pGL4.32 (in which NF-κB response elements drive transcription of the firefly luciferase reporter gene) and pRL-CMV (in which the CMV promoter drives expression of the Renilla luciferase reporter gene) vectors was transiently co-transfected into HEK293T cells. 48 hours later, the transfectants were exposed to individual compounds at a concentration of 20 μM for 2 hours, and then 100 ng/mL lymphotoxin-α1β2 (LT-α1β2) for 4 hours. LT-α1β2 selectively activates RelB/p52 and induces DNA-binding at NF-κB response elements^18^. The lymphotoxin-beta receptor (LTβR) is constitutively expressed in HEK293T cells^19^. DMSO was used as a control. Experiments were performed in triplicate for each compound. The percentage inhibition was derived with use of the formula: [1-(mean FL activity of triplicates of individual inhibitor/mean RL activity of triplicates of individual inhibitor)/(mean FL activity of triplicates of vehicle/mean RL activity of triplicates of vehicle)] × 100. Supplementary Table 1 lists the 58 small molecule inhibitors that were identified based on inhibition of ≥90% LT-α1β2-induced NF-κB (RelB/p52)/FL activity at 20 μM of the candidate drug.

To assess whether the drugs identified in the initial screening were specific to the non-canonical NF-κB pathway or pan-NF-κB activity inhibitors, we took advantage of a similar strategy as described in the initial screening. In this experiment, HEK293T cells were treated with 12-*O*-tetradecanoylphorbol-13-acetate (TPA) at 10 ng/mL (Fig. 1B). TPA selectively induces canonical NF-κB signaling and induces nuclear translocation of RelA/p50 heterodimers^20^. As shown in Table 1, 14 of 58 drugs identified as inhibitors of the non-canonical NF-κB pathway did not demonstrate activity against the canonical pathway and were studied further because of their more selective activity against the target pathway.

**Table 1:** Candidate inhibitors of LT-α1β2- but not TPA-induced NF-κB activity.

Candidates	Code
Pheniramine maleate	PM
Minaprine dihydrochloride	MD
Cyproheptadine hydrochloride	CH
Verapamyl hydrochloride	VH
Flutamide	FT
Nimesulide	NS
Alprenolol hydrochloride	AH
Deferoxamine mesylate	DM
Sulfapyridine	SP
Meglumine	MG
Tyloxapol	TX
Megestrol acetate	MC
(R)-Propranolol hydrochloride	PH

Modulation of kinase activity is an active field of study in the pharmaceutical industry because of the role of kinases in cellular function and communication. There are at least 4 different kinases combined in various oligomers involved in activation of NF-κB; trimeric IKKα/IKKβ/NEMO for the canonical pathway, and NIK-IKKα/IKKα dimers for the non-canonical pathway. We investigated whether the 14 selective non-canonical NF-κB inhibitors disrupted NIK and/or IKKα activity. We also assessed activity against IKKβ(which activates the canonical pathway) as a control. HEK293T cells were transfected with a mammalian expression vector for 48 hours, allowing ectopic expression of individual kinases, prior to being treated with the individual chemical compound and in turn LT-αα1β2 (for NIK and IKKα) or TPA (for IKKβ) (Fig. 1C). Wedelolactone, a validated IKKα inhibitor^21^, and staurosporine, a pan-kinase inhibitor^22^, were used as the positive controls. In Supplementary Table 2, we demonstrate that 7 drugs inhibited IKKα based on IKKα overexpression-induced de-repression of FL activity, compared with that transfected with an empty vector (p < 0.05). 3 drugs inhibited NIK based on NIK overexpression-induced de-repression of FL activity (p < 0.05). 2 agents, sulfapyradine and propranolol hydrochloride, inhibited both IKKα and NIK. None of the 14 drugs had a significant effect on FL activity when HEK293T that over-expressed IKKβ were stimulated with TPA (p > 0.05). As expected, staurosporine inhibited all kinase activity, and wedelolactone selectively inhibited IKKα and IKKβ.

### Effects of candidate inhibitors on expression of CRH and COX-2 in human placenta

To examine whether these chemical compounds are capable of inhibiting CRH and COX-2, we treated primary cultures of cytotrophoblast from term human placentas with the each of the 14 selective non-canonical NF-κB pathway inhibitors separately at a concentration of 20 μM for 24 hours. Western blot assays were used to assess the effects of each selective non-canonical NF-κB pathway inhibitor against CRH and COX-2. As shown in Fig. 1D, Supplementary Fig. 1, and Supplementary Table 3, 12 of the 14 drugs effectively repressed expression of both CRH and COX-2 in the human placenta.

### Effects of candidate inhibitors on efflux transporter MRP1 and cell viability of term human cytotrophoblast

**Figure 2:**
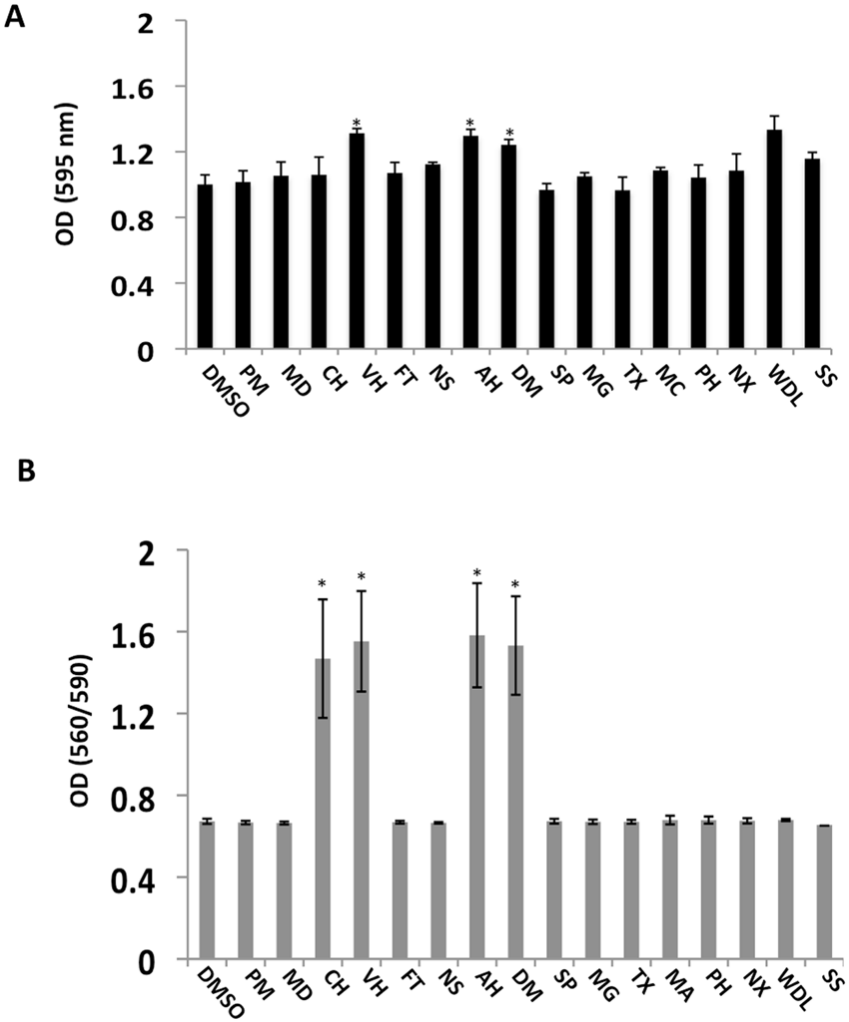
Assays of efflux transporter MRP1 and cell viability. (**A**) This ATPase assay was performed in the basic way as detailed in Materials and Methods. The bars indicate the average of OD (595 nm) against each individual hit with error bars representing the standard deviation from experiments performed in 3 independent term placentas. *p < 0.01. (**B**) Primary term cytotrophoblast were treated with the individual compound and cell viability was measured with use of the CellTiter-Blue cell viability assay kit (Promega). The bars indicate the average ration of OD 560/590 (nm) against each individual hit with error bars representing the standard deviation from experiments performed in 3 independent term placentas. *p < 0.01.

Drug efflux transporters in the human placenta can significantly influence the maternal-fetal transfer of a diverse array of drugs and other xenobiotics, which are exemplified by ATP binding cassette protein C1 or multidrug resistance-associated protein 1 (MRP1). MRP1 has been found to localize on the basal membranes of human term placental trophoblast^23^. It is a unidirectional efflux transporter, and the major roles of MRP1 include efflux of xenobiotic and endogenous metabolites, transport of inflammatory mediators, and development of drug resistance in a variety of diseases^24,25^. To determine whether the candidate drugs were potential substrates of MRP1, we incubated primary term cytotrophoblast with the individual compound at 20 μM for 24 hours and then assessed efflux by measuring ATP hydrolysis with use of an ATPase Assay Kit according to the MRP1 protocol (Solvo Biotechnology). As shown in Fig. 2A, 3 of the 14 drugs, verapamyl, alprenolol, and deferoxamine mesylate demonstrated significantly higher ATP hydrolysis, indicating that they may be substrates of MRP1. These agents would be lesser candidates for further study.

To investigate toxicity of the hits on human placenta, we incubated primary cytotrophoblast with each individual compound at 20 μM for 24 hours and measured the number of viable cells with use of the CellTiter-Blue cell viability assay kit (Promega). As shown in Fig. 2B, 4 of 14 drugs, cyproheptadine, verapamil, alprenolol, and deferoxamine mesylate were toxic to the human term placenta.

### NIK co-localizes with sulfapyridine and propranolol

**Figure 3:**
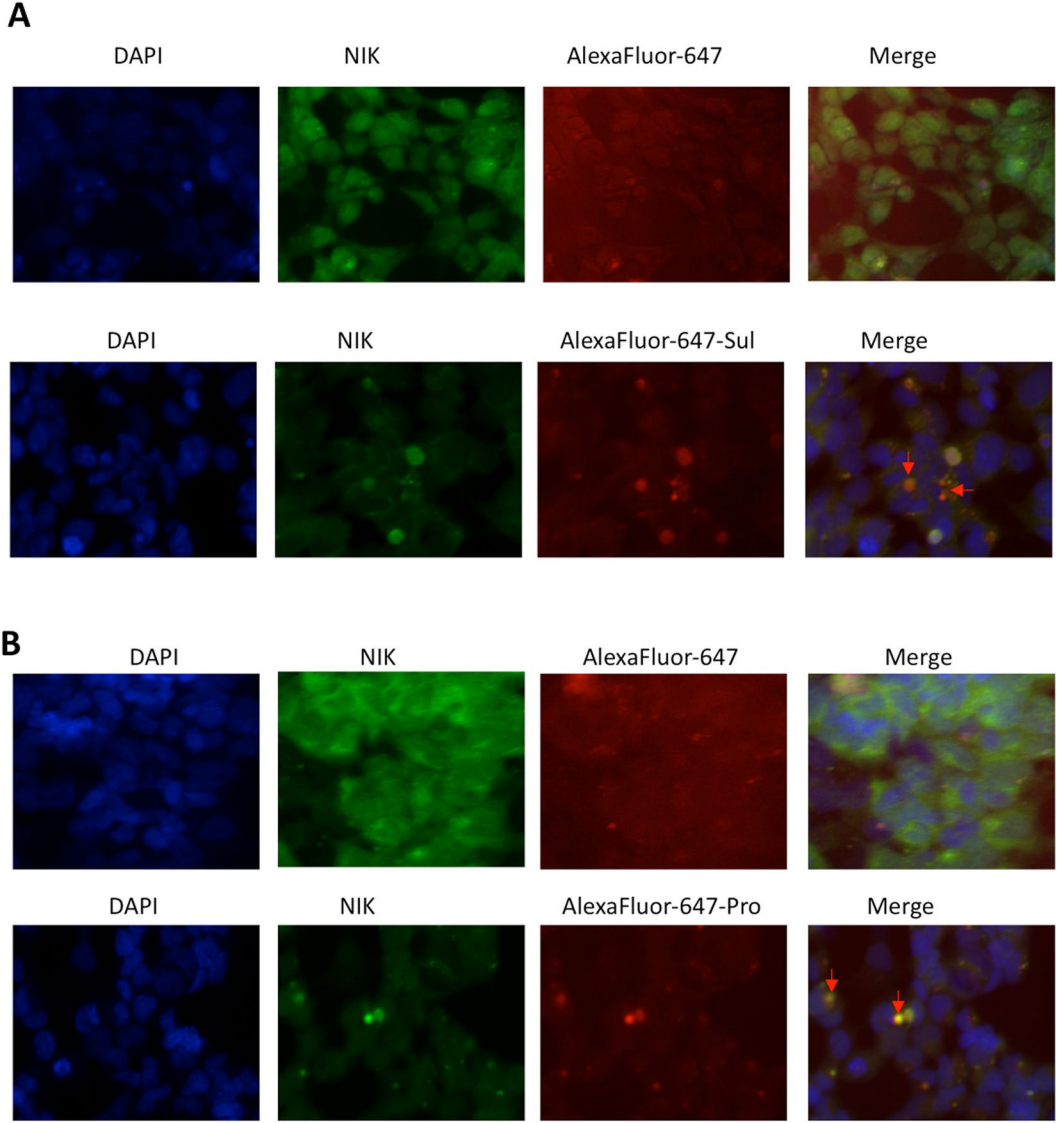
Co-localization of NIK and sulfapyridine or propranolol in HEK293 cell. (**A**) HEK293 cells were treated with Alexa Fluor 647 cadaverine (AlexaFluor-647) or Alexa Fluor 647 cadaverine-labeled sulfapyridine (AlexaFluor-647-Sul) for 24 hr, followed by IF staining. (**B**) HEK293 cells were treated with AlexaFluor-647 or Alexa Fluor 647 cadaverine-labeled propranolol (AlexaFluor-647-Pro) for 24 hr, followed by IF staining. Each experiment was repeated three times. Arrows indicate co-localization.

Sulfapyridine and propranolol were perhaps the most interesting candidate drugs because they selectively inhibited both NIK and IKKα, were not substrates of drug transporter MRP1, and they were not toxic to cytotrophoblast, so we chose to assess these agents further. To investigate whether they co-localized with NIK, we treated HEK293T cells with fluorescently (Alexa Fluor 647 cadaverine) labeled drug, which were subsequently subjected to immunofluorescence (IF) staining. As expected, we did not find any co-localization between cadaverine and NIK. In contrast, a strong co-localization between NIK and cadaverine-labeled sulfapyridine or propranolol was observed, which was confirmed by repeated experiments (Fig. 3).

We modeled docking of NIK (PDB accession code: 4IDV) and select candidate ligands (Supplementary Fig. 2). The GOLD docking results have shown an increased binding affinity of sulfapyridine and propranolol versus the controls including ZINC-1601221, previously described as a specific inhibitor to NIK^26^.

Taken together, these data strongly support that sulfapyridine or propranolol inhibits NIK activity by direct interaction.

### Dose-dependent effects of sulfapyridine or propranolol on expression of COX-2 and CRH

**Figure 4:**
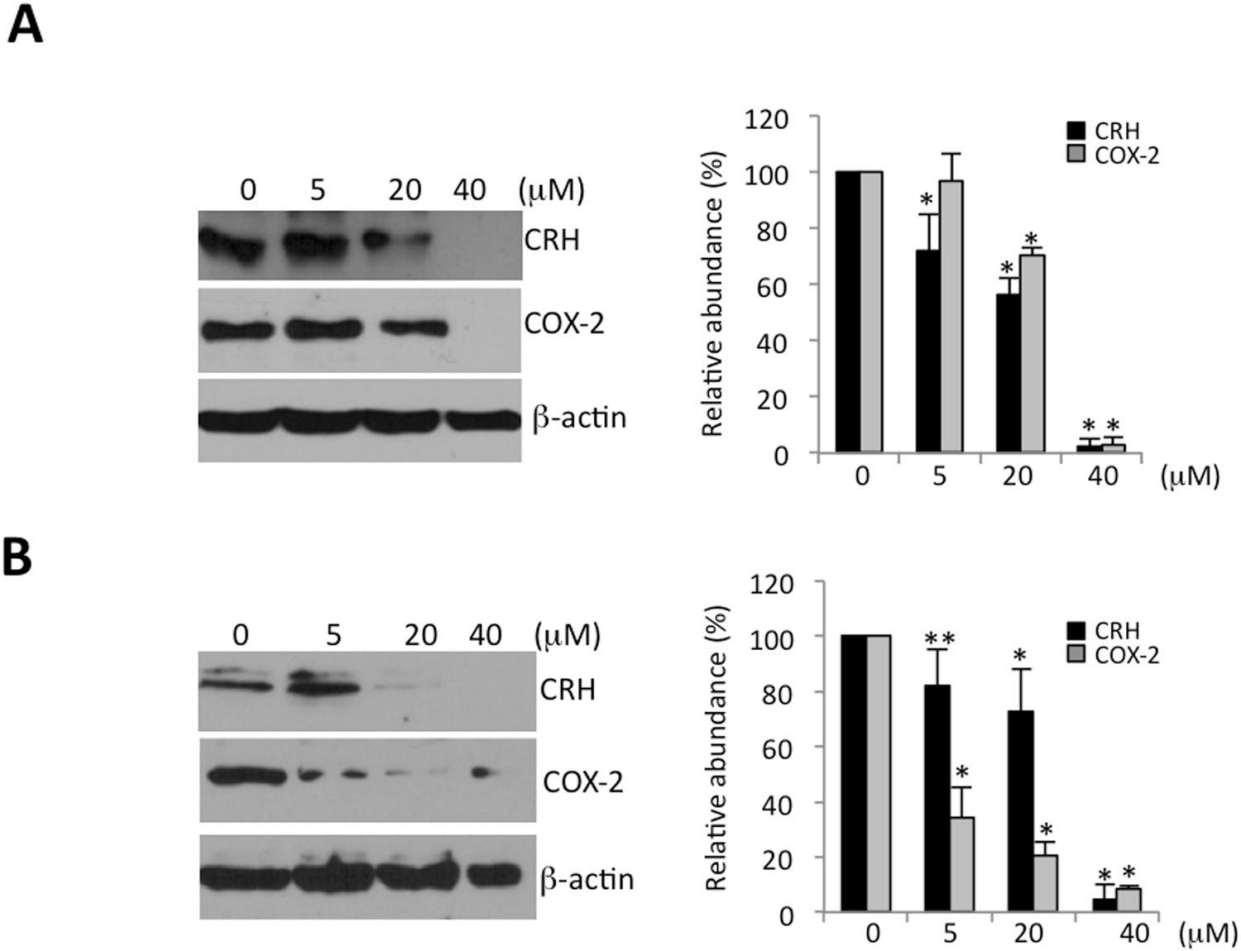
Dose-dependent effects of sulfapyridine or propranolol on COX-2 and CRH in primary cytotrophoblast. Human primary cytotrophoblast were treated with sulfapyridine (**A**) or propranolol (**B**) at concentrations as indicated for 24 hrs. The total cell lysates were subjected to Western blot analysis (N = 3 individual experiments). The full-length blots are shown in Supplementary Fig. 3. Western blot results were quantified with use of ImageJ (right panels). The relative protein levels of CRH or COX-2 treated with each drug at different concentrations relative to non-treatment (0) with normalization to β-actin were derived from the following formula: (peak area of CRH or COX-2 of each concentration)/(peak area of β-actin of the same lane) of/(peak area of CRH or COX-2 of non-treatment)/(peak area of β-actin of the same lane) × 100%. The relative abundance of CRH or COX-2 in the lane of non-treatment was considered 100%. *p < 0.01, **p < 0.05.

We performed a dose-response experiment for sulfapyridine and propranolol against the pro-labor genes COX-2 and CRH in trophoblast and used Western blot analysis to assess the candidate drug effects on protein levels. Figure 4 and Supplementary Fig. 3 demonstrate that both COX-2 and CRH protein levels gradually decreased as the drug concentrations increased.

## Discussion

We have shown that the non-canonical NF-κB signaling pathway positively regulates pro-labor genes of human placental origin including CRH and COX-2^12,27^, and may be a central regulator of the clock that governs length of pregnancy. As such, inhibition of this pathway is a target for drug therapy to modulate the placental clock, and can serve as a basis to develop new therapies to prevent spontaneous preterm birth.

In the current study, we screened 1,120 small molecule inhibitors and found 58 agents that had activity against the non-canonical NF-κB pathway. 14 of these 58 drugs selectively inhibited the non-canonical pathway and not the canonical NF-κB pathway. 12 of these 14 drugs also reduced abundance of CRH and COX-2 in cultures of cytotrophoblast from term human placentas. Sulfapyridine and propranolol appeared to inhibit NIK and IKKα, 2 essential kinases in the non-canonical pathway, while having no effect on IKKβ,a key mediator of the canonical pathway. Pheniramine maleate selectively inhibited NIK, while flutamide, tyloxapol, and nifuroxazide only had activity against IKKα. Drugs that inhibit both NIK and IKKα might have the greatest activity against the non-canonical pathway in animal studies, but because the role of non-canonical signaling in fetal development is not well characterized, selective NIK or IKKα inhibitors could have fewer unintended consequences, and as such, remain drugs of interest. None of these six agents (sulfapyridine, propranolol, pheniramine, flutamide, tyloxapol, or nifuroxide) was identified to be toxic to cytotrophoblast, and none was a substrate of the MRP1 transporter, so they could have activity on placental function *in vivo*. Additional *in vitro* studies to assess effects on other placental efflux transporters (e.g. BCRP1) would be required before determining which drugs might be studied in animal models.

Among these six drugs, sulfapyridine may be the most interesting candidate for further study. Sulfapyridine is a short-acting sulfonamide antibiotic and a metabolite of the non-steroidal anti-inflammatory drug sulfasalazine, which is safe for use during pregnancy and unlikely to increase the fetal morbidity or mortality^28^. It is readily absorbed orally and does not appear to be teratogenic or cause other harmful fetal effects. Interestingly in our study, and consistent with previous reports^29^, sulfasalazine inhibited the canonical NF-κB pathway in addition to the non-canonical pathway, while its metabolite sulfapyridine selectively inhibited only the non-canonical pathway.

In a 2007 Danish study of women with Crohn’s disease, women treated with sulfasalazine had larger babies than women with Crohn’s who did not require medication or those treated with azathioprine, prednisone, or both because of fewer preterm births in the sulfasalazine treated women. In fact, none of the 179 women treated with sulfasalazine delivered prior to 34 weeks gestational age, while women in the non-treated and azathioprine/prednisone groups delivered as early as 27 weeks of gestation^30^. Similarly, a 1981 United States study of the effects of inflammatory bowel disease on pregnancy found that women treated with sulfasalazine had lower preterm birth rates than women in the general population. These data from population studies add to the hypothesis that sulfapyridine may be potentially useful in preventing some cases of preterm birth.

Propranolol, a beta-adrenergic blocker, and pheniramine maleate, an antihistamine, may be worth further study, but both have a weak association with adverse pregnancy outcomes^31,32^. The finding that a beta-blocker could slow the placenta clock is interesting given that clinicians have used beta-adrenergic agonists to tocolyze preterm labor^33^. The target of beta-mimetic therapy for tocolysis is the beta-adrenergic on the receptor on the uterus, and not the placenta. Beta-mimetics are among the least effective therapies to prolong pregnancy in women with preterm labor^34^, perhaps because of the opposing effects on the uterus and placenta. Further characterization of the effects of propranolol and pheniramine to prevent preterm birth *in vivo* might serve as a basis for future drug design. Unlike sulfasalazine, we were unable to find any previous studies linking these two agents to a reduced incidence of preterm delivery.

Flutamide, tyloxapol, and nifuroxazide are poor candidates to prevent preterm birth for different reasons. Flutamide is a nonsteroidal antiandrogen used in the treatment of prostate cancer. Administration during pregnancy resulted in adverse effects on male genital development in experimental animals^35^. Tyloxapol is administered by nebulizer to aid in removal of bronchopulmonary secretions and human systemic absorption has not been characterized. Nifuroxazide has poor oral absorption and other drugs of the nitrofurans family are known to be substrates of the BCRP transporter^36^ and are actively effluxed from the placenta.

It should be noted that, of the 14 inhibitors that selectively blocked non-canonical NF-κB activity, 6 had no activity against NIK or IKKα, and 4 were toxic to cytorophoblast. But nimesulide, meglumine, and megestrol acetate inhibited CRH and COX-2 and were not cytotoxic. The non-canonical NF-κB pathway is regulated at multiple steps, such as proteasomal degradation of tumor necrosis factor receptor-associated factor-3 (TRAF3) or at the C-terminal of p100 to generate p52^14^. We suspect that the non-toxic drugs that had activity against the non-canonical pathway and inhibited CRH and COX-2 in cytotrophoblasts, but did not alter NIK or IKKα inhibited the proteasome at another step in activation of the non-canonical NF-κB pathway. Because less is known about the mechanisms of action of these agents against the target pathway, they would be of more interest for *in vivo* assessment when their mechanism of action is further characterized.

Interestingly, none of the drugs that inhibited IKKα blocked TPA-induced NF-κB activation (RelA/p50), even though IKKα is involved in signaling activation of the canonical NF-κB pathway. These results suggest that inhibition of IKKα alone in the trimeric IKKβ/IKKα/NEMO complex may not be sufficient to alter activation of the canonical NF-κB pathway in these systems. We also observed that treatment with minaprine or deferoxamine blocked non-canonical NF-κB signaling in HEK293T cells, but did not reduce expression of CRH and COX-2 in primary culture of cytotrophoblast. This could be a result of differences in the biology of HEK293T cells and cytotrophoblast. For example, these agents could be a substrate of a placental efflux transporter(s) other than MRP1 such as BCRP^37^.

These data could have implications outside of our study of non-canonical signaling in the placenta. For example, aberrant non-canonical NF-κB activation has been linked to multiple myeloma^38,39^ and other cancers, and the agents characterized here could have activity against some malignancies. Indeed, propranolol has been found to reduce lifetime cancer risk in several studies^40–42^. This effect has been presumably mediated by the beta-adrenergic receptor, but the data we have presented here suggests that this effect could also be mediated through the non-canonical NF-κκB pathway. The role of the non-canonical NF-κB pathway in other disease states is being actively investigated^14^.

## Methods

### Study approval

All patients signed a written informed consent for their specimen to be used for this study in a protocol approved by the Institutional Review Board of Rutgers Robert Wood Johnson Medical School and all methods were performed in accordance with the relevant guidelines and regulations.

### Primary term cytotrophoblast cell culture

Human placentas between 37 and 41 weeks estimated gestational age were collected with informed consent. Inclusion criteria were healthy women between the ages 18 to 45 years with a singleton gestation harvested during a cesarean section prior to the onset of labor. Purification of primary term cytotrophoblast was performed as recently described^10,12,43^. Cells were cultured in DMEM media containing 10% FBS at 37 °C and 5% CO_2_ for ≥24 hours prior to experimental conditions.

### Small molecule chemical library and reagents

The Prestwick Chemical Library, containing 1,120 small molecule chemical compounds, was obtained from The Cellular Screening Center, University of Chicago. pGL4.32 vector containing 5 copies of an NF-κB response element (NF-κB-RE) that drives transcription of firefly luciferase (FL) reporter gene, pRL-CMV vector containing CMV promoter that drives expression of *Renilla* luciferase (RL) reporter gene, and Dual-Glo Luciferase Assay System were purchased from Promega, Madison, WI. We used plasmids pUNO-hIKKα (InvivoGen, San Diego, CA), pCMV4-HA-NIK (Addgene, Cambridge, MA), and pRK7-IKK2/IKKb (Addgene) for ectopic expression of IKKα, NIK, and IKKβ in HEK293T cells, respectively. Antibodies used in Western blot assays included anti-CRH (Abnova, CA) and anti-COX-2 (Cell Signaling, Danvers, MA).

### Transfections of plasmid DNAs

HEK293T cell line (ATCC, USA) were cultured at 37 °C and 5% CO_2_ in 96-well plates. Plasmid DNA transfection was performed when cells reached confluence. For each transfection, we first incubated 0.5 μL of Lipofectamine 2000 (Invitrogen) with 0.1 μg of plasmid DNA vector as specified in 25 μL of serum-free media for 30 min at room temperature. The transfection mix was then transferred into each well.

### Dual-luciferase assay

HEK 293 T cells were lysed with 1x passive buffer and dual luciferase assays were performed according to the manufacturer’s protocol (Promega). Briefly, FL activity was measured by adding LAR I (30 μL) into one well of a 96-well white plate (PerkinElmer, Waltham, MA) and read in a Victor^3^ V Microplate Reader (PerkinElmer) for 5 sec. RL activity was measured by adding Stop & Glo (30 μL, Promega) to each well and reread for 5 sec.

### Western blot analysis

The total lysates were resuspended in 1x SDS loading buffer, boiled at 95 °C for 5 min, and centrifuged. Supernatants were resolved on SDS–10% PAGE and transferred onto PVDF membranes (Bio-Rad). Membranes were blocked in 5% nonfat milk powder in Phosphate Buffered Saline Tween (PBST) (10 mM phosphate buffer, pH 7.2; 150 mM NaCl; and 0.1% Tween 20) for 60 min, washed twice with PBST, and incubated with antibodies as indicated in 1% nonfat milk powder–PBST 20 at 4 °C overnight. The blots were probed with β-Actin antibody (Sigma) as the loading control. Membranes were washed 3 times with PBST, incubated with horseradish peroxidase-conjugated secondary antibodies (Jackson ImmunoResearch) at 1:5,000 in 1% nonfat milk powder–PBST, and developed by Immun-Star HRP Substrate (Bio-Rad). The Western blot results were quantified with use of ImageJ program.

### Efflux transporter assay

To assess whether the tested chemicals may be substrates of efflux transporters in the human placenta, we used the PREDEASY-MRP1 ATPase assay kit (Solvo Biotechnology, Cambridge, MA). Primary cytotrophoblasts were seeded in a 96-well plate at approximately 1 × 10^4^ cells/per well and cultured for 24 hours. Cells were then treated with the individual chemical compound (20 μM) with DMSO as the vehicle control for an additional 24 hours. ATPase assay was performed in duplicates according to the manufacturer’s protocol with the OD read at 595 nm in a microplate reader (Bio-Rad, CA).

### Cell viability assay

To examine effects of small molecule chemical compounds on viability of placental trophoblast, we used the CellTiter-Blue Cell Viability Assay kit (Promega), which provides a homogeneous, fluorometric method for estimating the number of viable cells present in multiwell plates. Primary term trophoblast (~1 × 10^4^ cells/per well in 96-well plate) were cultured for 24 hours and then treated with individual chemical compounds at a concentration of 20 μM for another 24 hours. Cell viability assay was performed according to the manufacturer’s instructions with OD read at both 560 nm and 590 nm in the microplate reader (Bio-Rad).

### Immunofluorescent staining

Sulfapyridine and propranolol were fluorescently labeled by first incubating 3 μL of each drug (20 μM) with 2 μL of N-Ethyl-N-(3-dimethylaminopropyl)carbodiimide hydrochloride (EDAC, Sigma-Aldrich) at 500 μM, 3 μL of Alexa Fluor 647 Cadaverine (Thermo Fisher Scientific), and 2 μL of water for 2 hrs at room temperature. Cytotrophoblast cells in 200 μL culture media were treated with 3 μL of this mixture or 1 μL Alexa Fluor 647 Cadaverine for 24 hrs. Then cells were fixed with 4% paraformaldehyde and permeabilized in 0.5% Triton X-100 in PBS at room temperature followed by incubation with primary antibody to NIK overnight at 4 °C and fluorophore-conjugated secondary antibodies (Alexa Fluor 488 goat anti-rabbit IgG, Invitrogen) for 1 hr at room temperature. The cells were counterstained with DAPI (Invitrogen) for 20 min at room temperature and visualized under a fluorescence microscope (Nikon, Japan).

### *In silico* evaluation of NIK inhibitors

The availability of the published structure of NIK domain co-crystallized in the Protein data bank (PDB) with a small molecule ligand enabled the investigation of the active site residues (20) utilizing molecular modeling and ligand receptor docking techniques. The scoring of binding was assessed using GOLD (Genetic Optimization for Ligand Docking) tool (21). Structural coordinates for the protein NIK was extracted from PDB structures 4IDV and optimized with MMFF94 force field and atomic charges, corrected, energy minimized and docked^44–46^. Water molecules were removed and hydrogen atoms were added to molecular structure of NIK before dockings were performed. The default algorithm speed was selected and number of poses for each ligand was set to 10. The ligand-binding site was defined as receptor site residues within 10 Å of the centroid of co-crystallized small molecule structure originally docked. The 10 individual binding poses were ranked according to GOLD scores. This process was performed in triplicate. Typically, the higher the GOLD score, the greater the binding affinity of the ligand for the receptor site.

### Statistical analysis

Each experiment was repeated a minimum of three times. We used one-way ANOVA and Student *t* test (one-tailed) to test significance among ≥ three and between two groups, respectively. P < 0.05 was considered statistically significant.

## Electronic supplementary material

Supplementary Tables & Figures

## References

[1] Matthews TJ, MacDorman MF (2013). Infant mortality statistics from the 2010 period linked birth/infant death data set. National vital statistics reports: from the Centers for Disease Control and Prevention, National Center for Health Statistics, National Vital Statistics System.

[2] Behrman, R. E., Butler, A. S. & Institute of Medicine (U.S.). Committee on Understanding Premature Birth and Assuring Healthy Outcomes. *Preterm birth: causes, consequences, and prevention*. (National Academies Press, 2007). 20669423

[3] Petraglia, F., Imperatore, A. & Challis, J. R. Neuroendocrine mechanisms in pregnancy and parturition. *Endocr Rev* **31**, 783–816, doi:er.2009-0019 (2010). 10.1210/er.2009-0019 20631004

[4] McLean M (1995). A placental clock controlling the length of human pregnancy. Nat Med.

[5] Wadhwa, P. D., Porto, M., Garite, T. J., Chicz-DeMet, A. & Sandman, C. A. Maternal corticotropin-releasing hormone levels in the early third trimester predict length of gestation in human pregnancy. *Am J Obstet Gynecol* **179**, 1079–1085, S0002937898702194 (1998). 10.1016/s0002-9378(98)70219-4 9790402

[6] Hobel, C. J., Dunkel-Schetter, C., Roesch, S. C., Castro, L. C. & Arora, C. P. Maternal plasma corticotropin-releasing hormone associated with stress at 20 weeks’ gestation in pregnancies ending in preterm delivery. *Am J Obstet Gynecol* **180**, S257–263, doi:S0002-9378(99)70712-X (1999). 10.1016/s0002-9378(99)70712-x 9914629

[7] Warren, W. B., Patrick, S. L. & Goland, R. S. Elevated maternal plasma corticotropin-releasing hormone levels in pregnancies complicated by preterm labor. *Am J Obstet Gynecol* **166**, 1198–1204; discussion 1204–1197 (1992). 10.1016/s0002-9378(11)90606-1 1566770

[8] Simhan, H. N. & Caritis, S. N. Prevention of preterm delivery. *N Engl J Med* **357**, 477–487, 357/5/477 (2007). 10.1056/NEJMra050435 17671256

[9] Challis JR (2002). Prostaglandins and mechanisms of preterm birth. Reproduction.

[10] Wang, B. *et al*. Glucocorticoid Receptor Signaling Contributes to Constitutive Activation of the Noncanonical NF-kappaB Pathway in Term Human Placenta. *Mol Endocrinol* **27**, 203–211, me.2012–1309 (2013). 10.1210/me.2012-1309 PMC5417329 23239753

[11] Wang, B., Parobchak, N., Rosen, M., Roche, N. & Rosen, T. Negative Effects of Progesterone Receptor Isoform-A on Human Placental Activity of the Noncanonical NF-kappaB Signaling. *J Clin Endocrinol Metab* **99**, E320–328, doi:jc.2013-2721 (2014). 10.1210/jc.2013-2721 24276461

[12] Wang, B., Parobchak, N. & Rosen, T. RelB/NF-kappaB2 Regulates Corticotropin-Releasing Hormone in the Human Placenta. *Mol Endocrinol* **26**, 1356–1369, doi:me.2012-1035 (2012). 10.1210/me.2012-1035 PMC5416986 22734038

[13] Di Stefano, V., Wang, B., Parobchak, N., Roche, N. & Rosen, T. RelB/p52-mediated NF-kappaB signaling alters histone acetylation to increase the abundance of corticotropin-releasing hormone in human placenta. *Sci Signal* **8**, ra85, 10.1126/scisignal.aaa9806 (2015). 10.1126/scisignal.aaa9806 26307012

[14] Sun, S. C. Non-canonical NF-kappaB signaling pathway. *Cell Res* **21**, 71–85, doi:cr2010177 (2011). 10.1038/cr.2010.177 PMC3193406 21173796

[15] Dejardin, E. The alternative NF-kappaB pathway from biochemistry to biology: pitfalls and promises for future drug development. *Biochem Pharmacol* **72**, 1161–1179, doi:S0006-2952(06)00500-4 (2006). 10.1016/j.bcp.2006.08.007 16970925

[16] Bhakar AL (2002). Constitutive nuclear factor-kappa B activity is required for central neuron survival. The Journal of neuroscience: the official journal of the Society for Neuroscience.

[17] Lai, T. S. *et al*. Identification of chemical inhibitors to human tissue transglutaminase by screening existing drug libraries. *Chem Biol* **15**, 969–978, doi:S1074-5521(08)00285-8 (2008). 10.1016/j.chembiol.2008.07.015 PMC2637080 18804034

[18] Mordmuller B, Krappmann D, Esen M, Wegener E, Scheidereit C (2003). Lymphotoxin and lipopolysaccharide induce NF-kappaB-p52 generation by a co-translational mechanism. EMBO Rep.

[19] Kuai J (2003). Endogenous association of TRAF2, TRAF3, cIAP1, and Smac with lymphotoxin beta receptor reveals a novel mechanism of apoptosis. J Biol Chem.

[20] Kang, M. I. *et al*. A selective small-molecule nuclear factor-kappaB inhibitor from a high-throughput cell-based assay for “activator protein-1 hits”. *Mol Cancer Ther* **8**, 571–581, doi:1535-7163.MCT-08-0811 (2009). 10.1158/1535-7163.MCT-08-0811 PMC2813146 19258426

[21] Kobori M (2004). Wedelolactone suppresses LPS-induced caspase-11 expression by directly inhibiting the IKK complex. Cell Death Differ.

[22] Ruegg UT, Burgess GM (1989). Staurosporine, K-252 and UCN-01: potent but nonspecific inhibitors of protein kinases. Trends Pharmacol Sci.

[23] Nagashige, M. *et al*. Basal membrane localization of MRP1 in human placental trophoblast. *Placenta* **24**, 951–958, doi:S014340040300170X (2003). 10.1016/s0143-4004(03)00170-x 14580377

[24] Keppler D, Leier I, Jedlitschky G (1997). Transport of glutathione conjugates and glucuronides by the multidrug resistance proteins MRP1 and MRP2. Biol Chem.

[25] Cole SP (2014). Targeting multidrug resistance protein 1 (MRP1, ABCC1): past, present, and future. Annu Rev Pharmacol Toxicol.

[26] Mortier, J. *et al*. NF-kappaB inducing kinase (NIK) inhibitors: identification of new scaffolds using virtual screening. *Bioorg Med Chem Lett* **20**, 4515–4520, doi:S0960-894X(10)00801-2 (2010). 10.1016/j.bmcl.2010.06.027 20580552

[27] Yu LJ, Wang B, Parobchak N, Roche N, Rosen T (2015). STAT3 cooperates with the non-canonical NF-kappaB signaling to regulate pro-labor genes in the human placenta. Placenta.

[28] Mogadam M, Dobbins WO, Korelitz BI, Ahmed SW (1981). Pregnancy in Inflammatory Bowel-Disease - Effect of Sulfasalazine and Corticosteroids on Fetal-Outcome. Gastroenterology.

[29] Wahl C, Liptay S, Adler G, Schmid RM (1998). Sulfasalazine: a potent and specific inhibitor of nuclear factor kappa B. The Journal of clinical investigation.

[30] Norgard B, Pedersen L, Christensen LA, Sorensen HT (2007). Therapeutic drug use in women with Crohn’s disease and birth outcomes: a Danish nationwide cohort study. Am J Gastroenterol.

[31] Pruyn SC, Phelan JP, Buchanan GC (1979). Long-term propranolol therapy in pregnancy: maternal and fetal outcome. American journal of obstetrics and gynecology.

[32] Gilboa SM (2009). Use of antihistamine medications during early pregnancy and isolated major malformations. Birth defects research. Part A, Clinical and molecular teratology.

[33] Canadian Preterm Labor Investigators, G (1992). Treatment of preterm labor with the beta-adrenergic agonist ritodrine. N Engl J Med.

[34] Haas DM, Caldwell DM, Kirkpatrick P, McIntosh JJ, Welton NJ (2012). Tocolytic therapy for preterm delivery: systematic review and network meta-analysis. BMJ.

[35] Foster PM, Harris MW (2005). Changes in androgen-mediated reproductive development in male rat offspring following exposure to a single oral dose of flutamide at different gestational ages. Toxicological sciences: an official journal of the Society of Toxicology.

[36] Feinshtein V (2010). Nitrofurantoin transport by placental choriocarcinoma JAr cells: involvement of BCRP, OATP2B1 and other MDR transporters. Archives of gynecology and obstetrics.

[37] Memon, N. *et al*. Regional expression of the BCRP/ABCG2 transporter in term human placentas. *Reprod Toxicol* **43**, 72–77, S0890-6238(13)00378-X (2014). 10.1016/j.reprotox.2013.11.003 PMC3946646 24269555

[38] Annunziata, C. M. *et al*. Frequent engagement of the classical and alternative NF-kappaB pathways by diverse genetic abnormalities in multiple myeloma. *Cancer Cell* **12**, 115–130, doi:S1535-6108(07)00203-6 (2007). 10.1016/j.ccr.2007.07.004 PMC2730509 17692804

[39] Keats JJ (2007). Promiscuous mutations activate the noncanonical NF-kappaB pathway in multiple myeloma. Cancer cell.

[40] Chang PY (2015). Propranolol Reduces Cancer Risk: A Population-Based Cohort Study. Medicine (Baltimore).

[41] Assimes TL, Elstein E, Langleben A, Suissa S (2008). Long-term use of antihypertensive drugs and risk of cancer. Pharmacoepidemiol Drug Saf.

[42] Monami M (2013). Further data on beta-blockers and cancer risk: observational study and meta-analysis of randomized clinical trials. Curr Med Res Opin.

[43] Tang Z (2011). Isolation of hofbauer cells from human term placentas with high yield and purity. Am J Reprod Immunol.

[44] Berman HM (2000). The Protein Data Bank. Nucleic acids research.

[45] Halgren TAB (1996). form, scope, parameterizaiton and performance of MMFF94. J. Comput Chem.

[46] Halgren TA (1996). Merck molecular force field. II. MMFF94 van der Waals and electrostatic parameters for intermolecular interactions J. Comput Chem.

