# Supplementary material for: Screening a small molecule library to identify inhibitors of NF-κB inducing kinase and pro-labor genes in human placenta
Source: Sci Rep. 2018 Jan 26;8:1657. doi: 10.1038/s41598-018-20147-0 (PMC5785954; doi:10.1038/s41598-018-20147-0)
Supplement: Supplementary file 1 — Supplementary Tables & Figures [file 41598_2018_20147_MOESM1_ESM.pdf]

TITLE

Screening a small molecule library to identify inhibitors of NF-κB inducing kinase and pro-labor genes in human placenta

Bingbing Wang[1,*], Nataliya Parobchak[1], Adriana Martin[1], Max Rosen[1], Lumeng Jenny Yu[1], Mary Nguyen[1,2], Kseniya Gololobova[1], and Todd Rosen[1,*]

1. Department of Obstetrics, Gynecology, and Reproductive Sciences, Division of Maternal-Fetal Medicine, Rutgers Robert Wood Johnson Medical School, New Brunswick, New Jersey, USA
2. Ernest Mario School of Pharmacy, Rutgers University, Piscataway, New Jersey, USA

Supplementary Table 1. Candidate inhibitors of LT-α1β2-induced NF-κB activity

**Candidates**	**MW**	**Candidates**	**MW**
Chloramphenicol	323	Trimethadione	141
Mefenamic acid	241	Sulfasalazine	398
Pheniramine maleate	240	Thiostrepton	1665
Diphenhydramine hydrochloride	292	Probenecid	285
Minaprine dihydrochloride	371	Tolazamide	311
Cyproheptadine hydrochloride	351	Nifuroxazide	275
Bromocryptine mesylate	750	Hydrastine hydrochloride	69
Verapamyl hydrochloride	455	Dimethisoquin hydrochloride	309
Nitrofurantoin	238	Dipivefrin hydrochloride	389
Alverine citrate	281	Citalopram Hydrobromide	324
Flutamide	276	Acacetin	284
Nimesulide	308	Pramoxine hydrochloride	293
Alprenolol hydrochloride	286	Deferoxamine mesylate	657
Ethisterone	312	Flecainide acetate	414
Indomethacin	358	Sulfapyridine	249
Mifepristone	430	Guanadrel sulfate	525
Diperodon hydrochloride	434	Hexylcaine hydrochloride	298
Dobutamine hydrochloride	338	Isocarboxazid	231
Enoxacin	320	Raloxifene hydrochloride	474
Colchicine	399	Meglumine	195
Debrisoquin sulfate	449	Chrysin	254
Etoposide	589	Tyloxapol	298
Prochlorperazine dimaleate	606	Megestrol acetate	385
Calciferol	397	Aminocaproic acid	131
Cyclosporin A	1203	Ribavirin	244
Gabazine	368	Letrozole	285
Niridazole	214	Moricizine hydrochloride	464
Piperine	285	(R)-Propranolol hydrochloride	259
Bromperidol	420	Ciprofibrate	289

Supplementary Table 2. Candidate inhibitors of kinases associated with NF-κB activation

**Candidates**	**IKKβ**	**IKKα**	**NIK**
Pheniramine maleate	–	–	+
Minaprine dihydrochloride	–	+	–
Cyproheptadine hydrochloride	–	–	–
Verapamyl hydrochloride	–	+	–
Flutamide	–	+	–
Nimesulide	–	–	–
Alprenolol hydrochloride	–	–	–
Deferoxamine mesylate	–	–	–
Sulfapyridine	–	+	+
Meglumine	–	–	–
Tyloxapol	–	+	–
Megestrol acetate	–	–	–
(R)-Propranolol hydrochloride	–	+	+
Nifuroxazide	–	+	–
Wedelolactone (WDL)	+	+	–/+
Staurosporine (SS)	+	+	+

Supplementary Table 3. Candidate inhibitors of CRH and COX-2 in human placenta

Candidates	CRH	COX-2
Pheniramine maleate	+	+
Minaprine dihydrochloride	+	–
Cyproheptadine hydrochloride	+	+
Verapamyl hydrochloride	+	+
Flutamide	+	+
Nimesulide	+	+
Alprenolol hydrochloride	+	+
Deferoxamine mesylate	–	–
Sulfapyridine	+	+
Meglumine	+	+
Tyloxapol	+	+
Megestrol acetate	+	+
(R)-Propranolol hydrochloride	+	+
Nifuroxazide	+	+
Staurosporine	+	+
Wedelolactone	+	+

Supplementary Figure 1

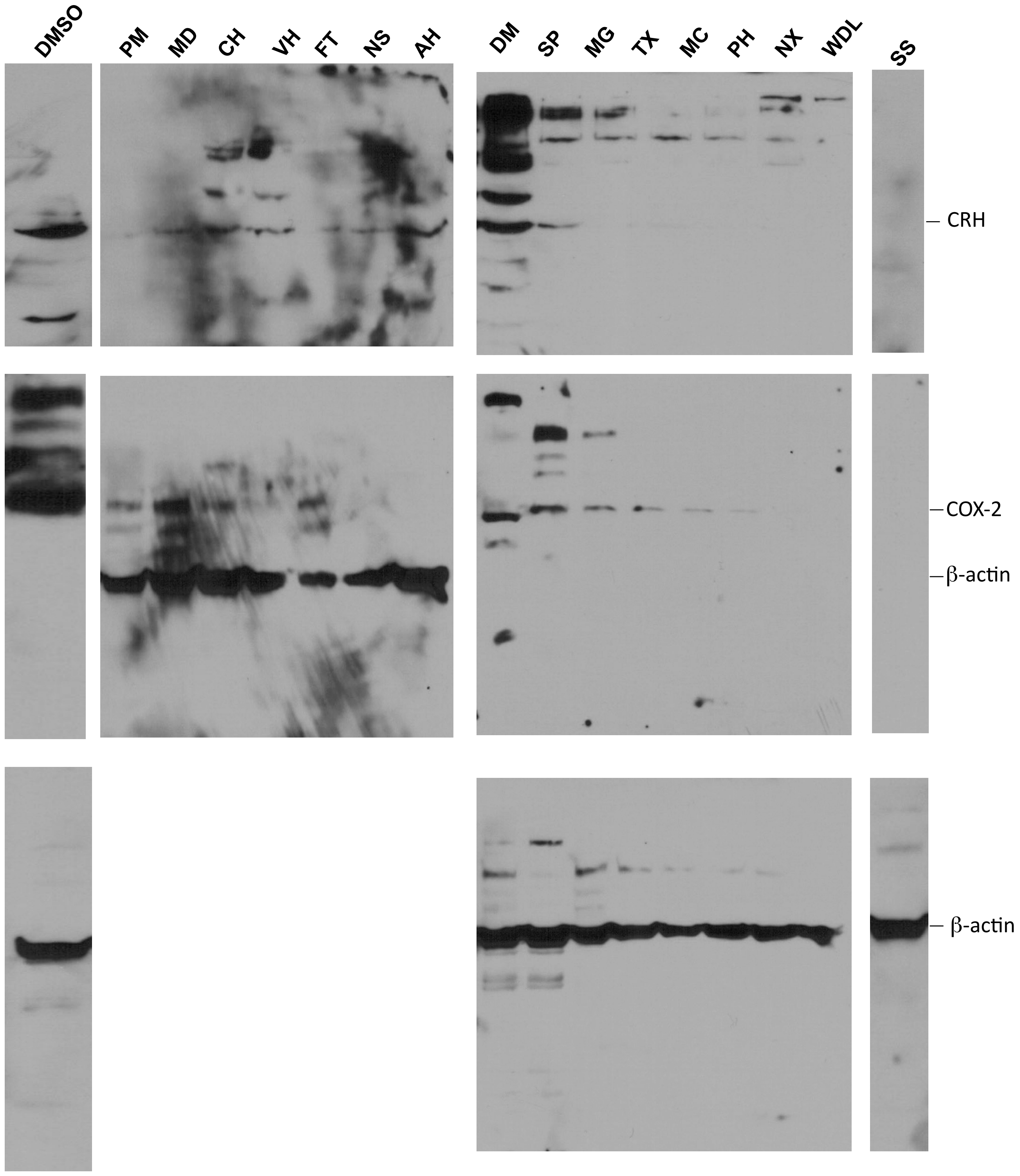

Supplementary Fig. 1. Full blots for Figure 1D**.**

Supplementary Figure 2

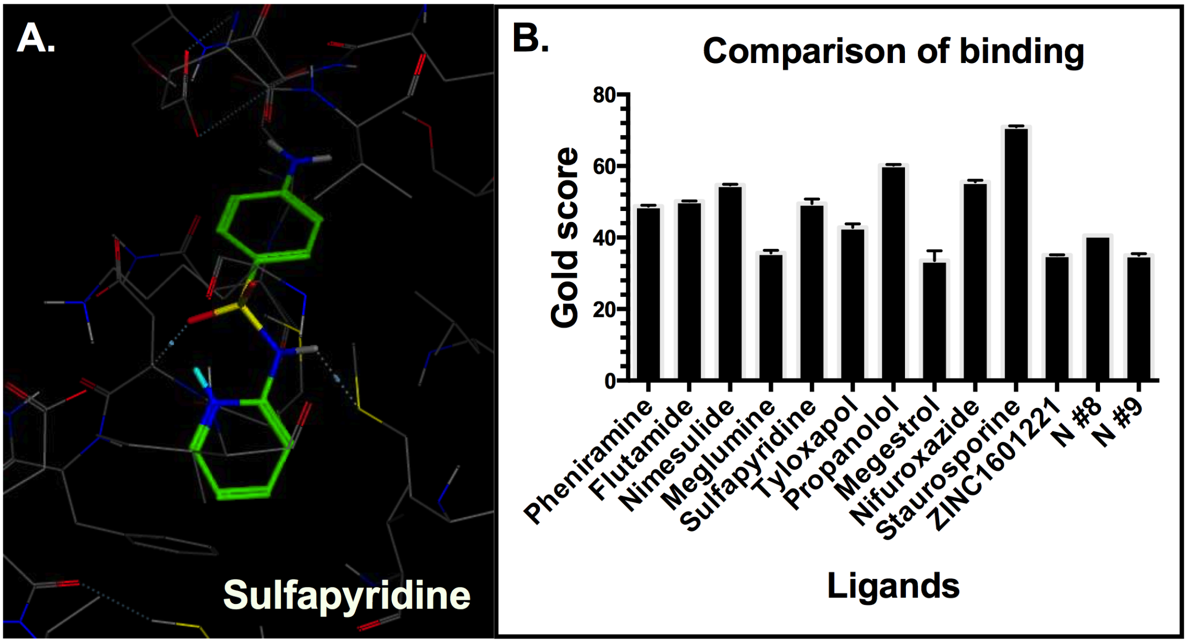

Supplementary Fig. 2. Molecular docking of ligands to NIK. (**A**) The pose of sulfapyridine to NIK. (**B**) GoldScores of screened inhibitors to NIK. The structures of the controls, ZINC1601221, N#8, and N#9 were obtained as previously depicted [30].

Supplementary Figure 3

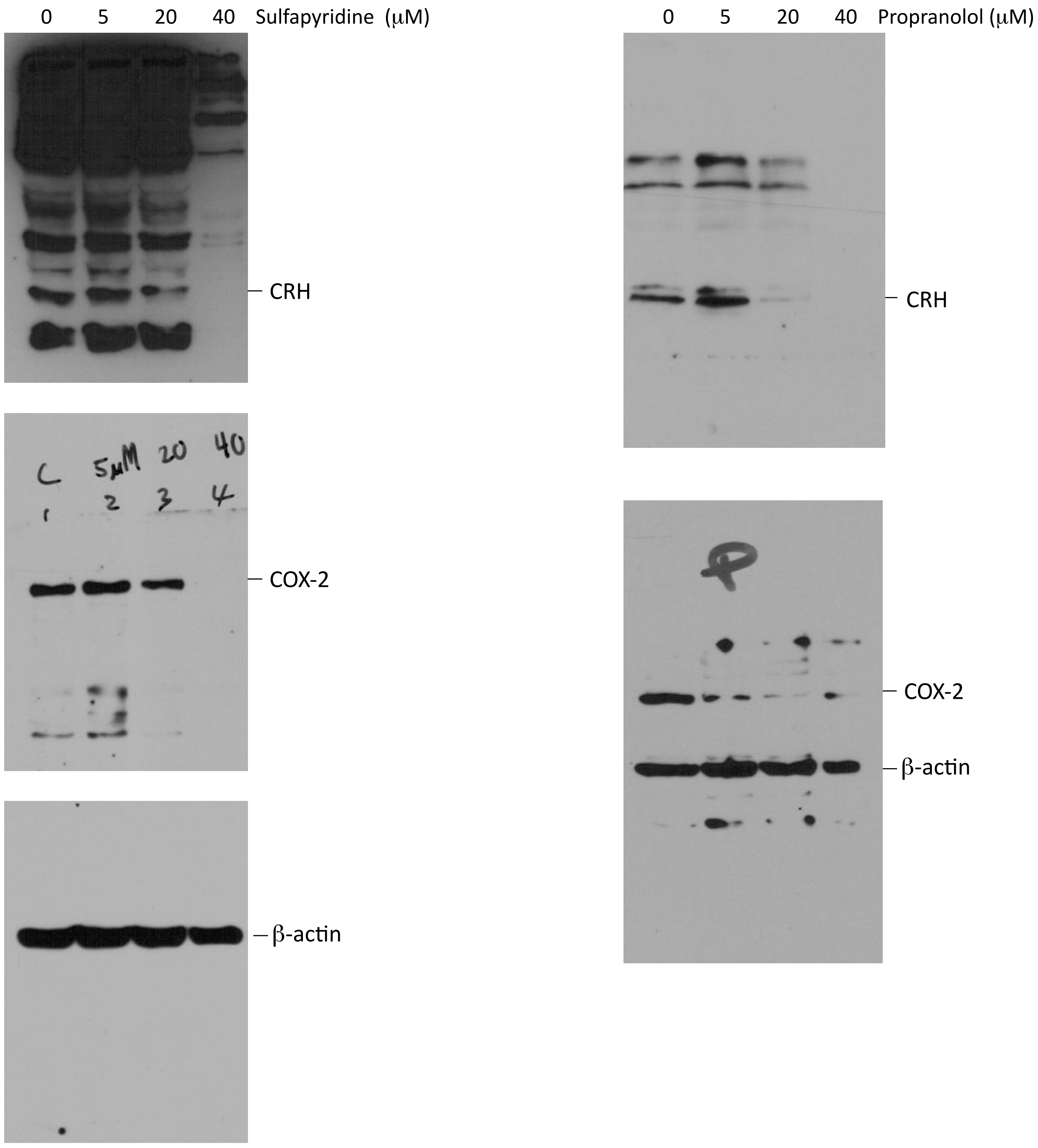

Supplementary Fig. 3. Full blots for Figure 4**.**
